# Cutaneous Adverse Events Associated with Interferon-β Treatment of Multiple Sclerosis

**DOI:** 10.3390/ijms160714951

**Published:** 2015-07-02

**Authors:** Annette Kolb-Mäurer, Matthias Goebeler, Mathias Mäurer

**Affiliations:** 1 Klinik und Poliklinik für Dermatologie, Venerologie und Allergologie, Universitätsklinikum Würzburg, Josef-Schneider-Str. 2, 97080 Würzburg, Germany; E-Mail: goebeler_m1@ukw.de; 2 Klinik für Neurologie, Caritas Krankenhaus Bad Mergentheim gGmbH, Uhlandstr. 7, 97980 Bad Mergentheim, Germany; E-Mail: Mathias.Maeurer@ckbm.de

**Keywords:** multiple sclerosis, interferon-β, therapy, cutaneous adverse events, psoriasis

## Abstract

Interferons are widely used platform therapies as disease-modifying treatment of patients with multiple sclerosis. Although interferons are usually safe and well tolerated, they frequently cause dermatological side effects. Here, we present a multiple sclerosis (MS) patient treated with interferon-β who developed new-onset psoriasis. Both her MS as well as her psoriasis finally responded to treatment with fumarates. This case illustrates that interferons not only cause local but also systemic adverse events of the skin. These systemic side effects might indicate that the Th17/IL-17 axis plays a prominent role in the immunopathogenesis of this individual case and that the autoimmune process might be deteriorated by further administration of interferons. In conclusion, we think that neurologists should be aware of systemic cutaneous side effects and have a closer look on interferon-associated skin lesions. Detection of psoriasiform lesions might indicate that interferons are probably not beneficial in the individual situation. We suggest that skin lesions may serve as biomarkers to allocate MS patients to adequate disease-modifying drugs.

## 1. Introduction

Multiple sclerosis (MS) is a chronic inflammatory disease of the central nervous system affecting mainly young adults. The disease is characterized by the relapsing occurrence of neurological deficits caused by a T cell-mediated autoimmune attack directed against the central nervous system tissue [1]. To date there is no cure for this chronic inflammatory disease; however, within recent years there has been huge progress in the development of anti-inflammatory drugs that modulate the disease course and reduce disability progression [2].

Approved first line therapies for the treatment of MS are different interferon-β (IFN-β) preparations, which require self-administration via subcutaneous (s.c.) or intramuscular (i.m.) injections [2]. Four IFN-β formulations are currently approved for the treatment of MS: IFN-β 1b (Betaseron^®^/Extavia^®^) injected every other day s.c., IFN-β 1a (Rebif^®^) injected three times a week s.c., IFN-β 1a (Avonex^®^) injected once a week i.m. and pegylated IFN-β 1a (Plegridy^®^) injected every two weeks s.c.

Interferons are known to affect multiple immunological processes resulting in an overall beneficial effect in MS but the exact mechanism of action is still elusive [3]. On the other hand, interferons are generally very safe and well tolerated, which makes them widely used drugs in MS. Side effects include flu-like symptoms, transient laboratory abnormalities, menstrual disorders, and increased spasticity [4]. Cutaneous adverse events are frequently reported by patients using interferons for the treatment of MS [5]. These include local reactions at the injection site with erythema, induration, swelling and pain. In addition to these local side effects persistent and systemic cutaneous complications may occur [4].

Our report presents a case of a patient developing new-onset psoriasis while on treatment with IFN-β and provides an overview on the frequency and spectrum of cutaneous adverse reactions associated with IFN treatment in MS patients.

## 2. Results and Discussion

Based on the case of a patient who developed psoriasis after initiation of IFN-β therapy we review skin manifestations developing during therapy with this immune-modulating agent and discuss potential consequences for IFN-β-based treatment strategies in MS patients.

### 2.1. Local Injection Site Reactions

**Figure 1:**
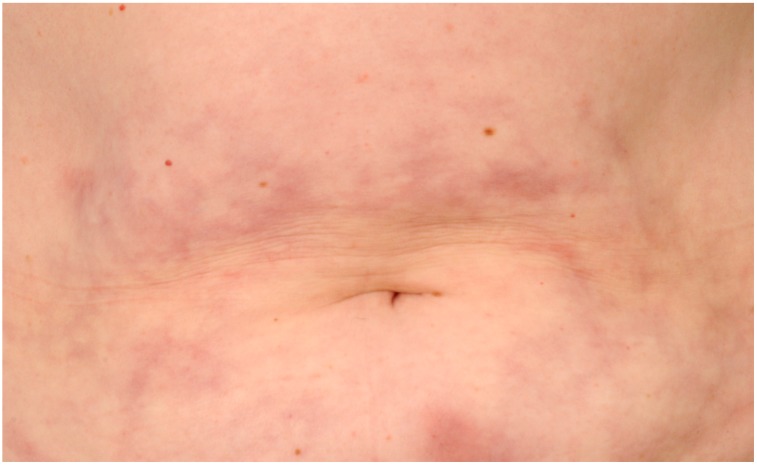
Characteristic injection site reactions in a patient injecting IFN-β s.c.

The most common cutaneous adverse events in MS patients using interferons are local injection site reactions. These include redness/erythema of the skin, bruises, eczema-like reactions, pain, pruritus, swelling and induration of the skin around injection sites (Figure 1). These symptoms usually appear shortly after starting interferon therapy, but may also appear several years after implementation of interferons [6]. Mild skin reactions occur in up to 90% of the patients using the s.c. application route. In comparison, only 33% of patients using i.m.-injected interferon complain of cutaneous adverse events [5]. Local injection site reactions are seen more frequently in areas with reduced subcutaneous fat tissue [4]. A wrong injection technique with injections being applied too superficially into the tissue (intradermal *vs.* subcutaneous injection) may also contribute to the occurrence of local site reactions.

IFN-β may trigger frequently observed inflammatory skin reactions through chemokine induction followed by immune cell extravasation. Skin biopsies from patients receiving IFN-β showed strong expression of the CCL2 and CXCL10 chemokines, facilitating trafficking of T cells from the circulation to sites of evolving skin lesions [7].

Compared to these very common adverse events that have been reported with high incidences in clinical trials, more severe cutaneous reactions such as deep ulcerations, skin infections or necrosis (1%–3%) are rare [5]. Histologies obtained from different cutaneous lesions showed perivascular lymphocytic infiltration, panniculitis and focal thrombosis as a function of severity of the local reaction [8]. The pathogenesis of skin ulcerations and necrosis, however, is still elusive [3]. Histopathology revealed thrombosis of dermal vessels, potentially due to an abnormal aggregation of platelets after injection of IFN-β [9]. In addition, local vasospasms have been discussed as underlying cause. Another assumption is that hypersensitivity to interferons may cause leukocytoclastic vasculitis [10].

Casoni *et al.* [11] described necrotizing skin lesions in patients injecting IFN-β 1b s.c. They observed an association between the occurrence of neutralizing antibodies to interferon and skin necrosis and assumed that an immune complex vasculitis may be the underlying cause of skin necrosis.

The occurrence of Nicolau syndrome (embolia cutis medicamentosa), a rare iatrogenic cutaneous reaction that usually occurs immediately after intramuscular drug injection, has been described in single patients after IFN-β 1a administration [12]. Moreover, lobar panniculitis with lipoatrophy has been reported after accidental s.c. injection of an i.m. IFN-β formulation [13].

After recovery post-inflammatory hyperpigmentation may persist. In addition, in some patients scars and lipoatrophy remain. To identify potential risk factors for the development of skin reactions different conditions have been examined, however, no associations were found with respect to preexisting atopic dermatitis, body mass index, gender or the usage of an autoinjector. However, there was a trend towards a higher occurrence of lipoatrophy in females [14].

To minimize cutaneous adverse events patients should receive a detailed introduction into injection techniques und precautions to avoid cutaneous adverse events. These include warming of the substance before injection, aseptic injection technique, regular change of the injection site and massage of the injection site right after application of the drug [15,16]. In case of erythema or eczema-like reactions the use of anti-inflammatory gels or topical steroids is recommended. In addition, halving of the diluent might also help to prevent cutaneous adverse events [17].

### 2.2. Systemic Cutaneous Adverse Events

Immune-mediated and inflammatory dermatological diseases in association with IFN-β treatment are generally rare. While other autoimmune diseases are associated with MS it is currently uncertain whether psoriasis occurs in MS patients with higher incidence [18]. However, Dobson and Giovannoni [19] recently performed a systematic review and calculated the overall risk for additional autoimmune diseases in patients with MS and their first-degree relatives. The odds ratio of thyroid disease was increased in both individuals with MS and their relatives. A similar association was seen between MS and inflammatory bowel disease and psoriasis, although not in relatives.

It has been reported that administration of IFN-β may cause exacerbation of cutaneous psoriasis [20]. The occurrence or recurrence of psoriasis may be related to the drug itself or to an increased susceptibility to autoimmune disorders in MS patients. Recently, Mantia and Capsoni published a case where they reported worsening of cutaneous psoriasis and activation of arthritis during IFN-β therapy [21]. Psoriasis resolved after withdrawal of IFN-β and upon subsequent azathioprine therapy. However, new-onset of psoriasis in a patient treated with IFN-β—as in our case—appears to be a very rare condition. López-Lerma *et al.* communicated a case in 2009 [22] describing a 37-year-old man without a personal or familial history of psoriasis who developed scaly and erythematous plaques on trunk and limb three month after starting IFN-β 1a (44µg, thrice weekly) for relapsing-remitting MS. The skin lesions disappeared after initiation of systemic steroid treatment for a relapse of MS.

Psoriasis is characterized by abnormal proliferation of keratinocytes in response to chronic inflammatory stimulation. Experiments using blocking antibodies against interferons have demonstrated that these cytokines play a pathogenic role in psoriasis [23]. IFN-β exacerbates Th17-mediated inflammatory disease. These findings could be a possible explanation for the initiation and maintenance of psoriasis in patients treated with IFN-β since the IL-23/Th17 axis is of major importance for the pathogenesis of psoriasis. Th17 cells mainly produce IL-17, which is responsible for the recruitment of neutrophilic granulocytes and therefore for the prevention of infections. IL-17 together with IL-23 can elicit psoriasis-like lesions when injected into normal skin [24]. In addition, affected skin and serum obtained from psoriasis patients show elevated levels of IL-17 that correlate with the severity of the disease. Furthermore, application of anti-IL-17 monoclonal antibodies is a promising treatment for psoriasis [25].

Interestingly, in murine experimental autoimmune encephalomyelitis (EAE) with prominent Th17 response IFN-β also results in an exacerbation of EAE [26]. These findings are well compatible with clinical studies suggesting that a prominent Th17 induction might result in a decreased efficacy or even worsening of MS patients treated with IFN-β [27].

Neuromyelitis optica (NMO) has some clinical similarities to MS; however, NMO lesions present with granulocytes including eosinophils and neutrophils. IL-17 and chemokines that recruit granulocytes to the CNS are elevated in serum. Interferons have been implicated as important mediators of NMO pathology. In clinical trials, IFN-β showed no benefit but exacerbations of this disease [27].

Furthermore, type I interferons play an important pathogenic role in systemic lupus erythematosus (SLE), which is characterized by high serum activity of interferons [27]. Arrue and co-workers observed lupus-like reactions at interferon injection sites in five patients receiving interferon—two of them obtained IFN-β treatment for multiple sclerosis [28]. These patients showed dense inflammatory infiltrates around the blood vessels, sweet glands and hair follicles with hydropic degeneration of follicular basal cells. However, anti-nuclear antibodies and related anti-DNA immunologic alterations were not detected.

Somani *et al.* reported on a 57-year-old MS patient with a new-onset of dermatomyositis, which developed during treatment with IFN-β [29]. Dermatomyositis and SLE share common histologic features and a similar gene expression signature with an overexpression of type I interferon-inducible genes. The patient had been treated for five years with IFN-β 1a (30 µg i.m.) once a week before he presented with violaceous skin eruptions involving his face, chest, back, upper extremities and knees. Some weeks later, he developed periorbital edema and proximal muscle weakness. Biopsy specimens were consistent with dermatomyositis and he improved after cessation of IFN-β. According to the clinical observation and results from *in vitro* studies the authors conclude that the symptoms seen in the patient were associated with IFN-β treatment and subsequent signaling events.

In animal models of SLE a blockade of interferons led to an amelioration of the disease while treatment with type I interferons was associated with increased disease activity [30]. Th17 cells also play a role in SLE, which is reflected by elevated numbers of Th17 cells and increased IL-17 levels in SLE patients. Autoimmune diseases that show deterioration when treated with type I interferon usually respond to TNF-α and IL-23 blockade, while MS patients are reported to worsen when exposed to anti-TNF-based treatment strategies [27].

Hügle *et al.* demonstrated the onset of systemic sclerosis (SSc) in three MS patients while on IFN-β treatment [31]. All patients were over 50 years of age and had received interferon treatment for several years before Raynaud’s phenomenon, sclerodactyly, myalgia and arthralgia occurred. The authors reviewed the literature and analyzed nine other cases of systemic sclerosis coinciding with MS. Of all twelve patients eight suffered from limited cutaneous SSc, three from diffuse cutaneous SSc and one patient from antisynthetase syndrome. Eleven patients developed SSc after onset of MS and manifested with skin sclerosis after a mean of 14.9 years. In five patients, IFN-β was commenced before the development of skin sclerosis with a range of 1–8 years. Ultimately, however, it remains unclear whether development of SSc was interferon-related in these patients.

Previously, it has been reported that interferon induces or worsens vitiligo. It is believed that vitiligo is induced via antimelanocyte autoantibodies or activiation of cytotoxic T-cells. In 2009 Kocer and co-workers reported the case of a 33-year-old female with MS treated with IFN-β 1a (22 µg s.c. three times weekly), who developed depigmented maculae on the dorsal aspects of her hands [32]. At that time point she had been on IFN-β treatment for two years. Twenty-two months after appearance of the first skin lesions new depigmented patches occurred on perioral regions and the chin. These vitiligo lesions significantly improved 3 months after cessation of IFN-β 1a and were stable on follow-up.

In 2012 Chakravarty *et al.* described the acute onset of rash and fever in a patient with MS after three years of continuous IFN-β treatment [33]. Skin biopsy showed the typical findings of sarcoidosis with non-caseating granulomas. The authors reviewed the literature and identified another four cases; they concluded that the development of sarcoidosis during treatment of MS with IFN-β represents an exceedingly rare event.

## 3. Case Report

A 25-year-old woman with a 1½ year history of relapsing-remitting MS presented with a new-onset of scaly and erythematous plaques on the abdomen and lower extremities. Twelve months earlier, she had started treatment with subcutaneous IFN-β 1a (Rebif^®^ 44 µg, thrice weekly). Six month after first administration inflammatory skin reactions appeared at the site of previous subcutaneous injections that subsequently transformed into psoriasiform plaques. Skin lesions spread out onto the lower extremities. There was no personal or family history of psoriasis.

**Figure 2:**
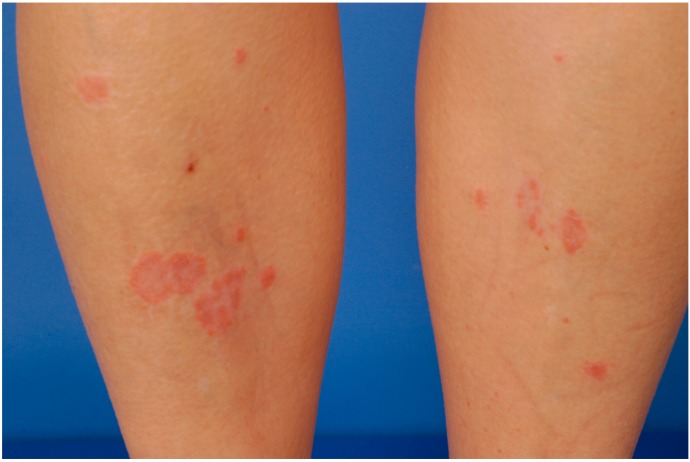
Scaly erythematous plaques occurring on the lower extremity 6 months after initiation of interferon-β (IFN-β).

**Figure 3:**
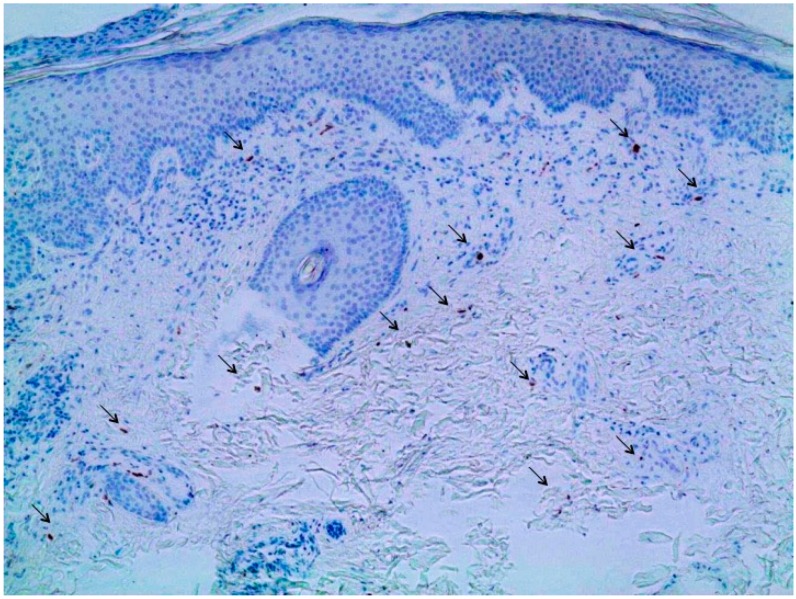
Immunohistochemistry of a skin biopsy obtained from the right thigh showing a mixed inflammatory infiltrate in the dermis with intermingled Interleukin 17 (IL17)-expressing cells (arrows). Furthermore, psoriasiform epidermal hyperplasia with hyperkeratosis and parakeratosis can be observed. Magnification 100-fold.

Clinical examination on presentation revealed multiple erythematous plaques distributed over the abdomen and lower extremities (Figure 2). Scaling plaques covered the elbows. A skin biopsy specimen showed psoriasiform epidermal hyperplasia with hyperkeratosis and parakeratosis. Neutrophilic extravasates were found in the upper epidermis and a perivascular inflammatory cell infiltrate in the papillary dermis with interspersed IL-17-positive cells (Figure 3).

Due to the suspicion that psoriasis might have been induced by IFN-β 1a (Rebif^®^ 44 µg) treatment, therapy was discontinued. The psoriatic lesions improved after withdrawal of IFN-β 1a (Rebif^®^ 44 µg) and upon addition of a topical Vitamin D analogue (calcipotriol). Finally, treatment with dimethylfumarate (480 mg daily dose, formula magistralis) was chosen as a therapeutic option for the treatment of both diseases. Treatment was well tolerated with only marginal gastrointestinal problems. After more than twelve months on treatment with fumarates, the patient’s psoriatic lesions completely disappeared and no new neurologic symptoms developed. Finally, her EDSS was 0 and MRI of the brain revealed no further evidence for disease progression.

## 4. Conclusions

Cutaneous adverse events are a common cause for non-adherence to disease-modifying treatments and might result in discontinuation of therapy [5,14,34]. Therefore, neurologists should be aware of cutaneous side effects in MS patients treated with interferons and should regularly inspect injection sites.

In contrast to local cutaneous side effects systemic cutaneous adverse events are rare. New-onset and exacerbation of psoriasis—as in our patient—has been previously reported in MS patients while on IFN-β treatment [22].

Unlike the beneficial effect in MS patients, IFN-β has a pro-inflammatory effect in psoriasis, where the IL-23/Th17 pathway plays a key role in pathogenesis.

With respect to the potential occurrence of Th17-mediated skin diseases in association with the application of type I interferons we suggest continuous monitoring of patients for psorasisform and lupus-like skin lesions while under treatment with interferons since this might indicate a shift to a Th17 immune response. In these patients skin lesions may serve as a biomarker for an inefficient response to immunomodulatory treatment with interferon; however, further studies are needed to validate this hypothesis. Accordingly, such patients should preferentially receive another disease-modifying drugs (DMD). Treatment with fumarates, which is known to induce IL-4-producing Th2 cells and to generate type II dendritic cells releasing IL-10 instead of IL-12 and IL-23 [35], may be an alternative approach in those patients. In addition, we believe that interferons should be avoided as first line DMDs in MS patients with a history of psoriasis.

## References

[1] Frohman E.M., Racke M.K., Raine C.S. (2006). Multiple sclerosis—The plaque and its pathogenesis. N. Engl. J. Med..

[2] Compston A., Coles A. (2002). Multiple sclerosis. Lancet.

[3] Dhib-Jalbut S., Marks S. (2010). Interferon-beta mechanisms of action in multiple sclerosis. Neurology.

[4] Walther E.U., Hohlfeld R. (1999). Multiple sclerosis: Side effects of interferon beta therapy and their management. Neurology.

[5] Balak D.M., Hengstman G.J., Çakmak A., Thio H.B. (2012). Cutaneous adverse events associated with disease-modifying treatment in multiple sclerosis: A systematic review. Mult. Scler..

[6] Gaines A.R., Varricchio F. (1998). Interferon beta-1b injection site reactions and necroses. Mult. Scler..

[7] Buttmann M., Goebeler M., Toksoy A., Schmid S., Graf W., Berberich-Siebelt F., Rieckmann P. (2005). Subcutaneous interferon-beta injections in patients with multiple sclerosis initiate inflammatory skin reactions by local chemokine induction. J. Neuroimmunol..

[8] Ohata U., Hara H., Yoshitake M., Terui T. (2010). Cutaneous reactions following subcutaneous beta-interferon-1b injection. J. Dermatol..

[9] Elgart G.W., Sheremata W., Ahn Y.S. (1997). Cutaneous reactions to recombinant human interferon beta-1b: The clinical and histologic spectrum. J. Am. Acad. Dermatol..

[10] Feldmann R., Löw-Weiser H., Duschet P., Gschnait F. (1997). Necrotizing cutaneous lesions caused by interferon beta injections in a patient with multiple sclerosis. Dermatology.

[11] Casoni F., Merelli E., Bedin R., Martella A., Cesinaro A., Bertolotto A. (2003). Necrotizing skin lesions and NABs development in a multiple sclerosis patient treated with IFNbeta 1b. Mult. Scler..

[12] Koontz D., Alshekhlee A. (2007). Embolia cutis medicamentosa following interferon beta injection. Mult. Scler..

[13] Weise G., Hupp M., Kerstan A., Buttmann M. (2012). Lobular panniculitis and lipoatrophy of the thighs with interferon-β1a for intramuscular injection in a patient with multiple sclerosis. J. Clin. Neurosci..

[14] Balak D.M., Hengstman G.J., Hajdarbegovic E., van den Brule R.J., Hupperts R.M., Thio H.B. (2013). Prevalence of cutaneous adverse events associated with long-term disease-modifying therapy and their impact on health-related quality of life in patients with multiple sclerosis: A cross-sectional study. BMC Neurol..

[15] Arnason B.G. (2005). Long-term experience with interferon beta-1b (Betaferon) in multiple sclerosis. J. Neurol..

[16] Nakamura Y., Kawachi Y., Furuta J., Otsuka F. (2008). Severe local skin reactions to interferon beta-1b in multiple sclerosis-improvement by deep subcutaneous injection. Eur. J. Dermatol..

[17] Zecca C., Yawalkar N., Gobbi C. (2012). Improvement of interferon-beta related skin reactions after diluent halving: first experience of five patients. Patient Prefer. Adher..

[18] Nielsen N.M., Frisch M., Rostgaard K., Wohlfahrt J., Hjalgrim H., Koch-Henriksen N., Melbye M., Westergaard T. (2008). Autoimmune diseases in patients with multiple sclerosis and their first-degree relatives: a nationwide cohort study in Denmark. Mult. Scler..

[19] Dobson R., Giovannoni G. (2013). Autoimmune disease in people with multiple sclerosis and their relatives: A systematic review and meta-analysis. J. Neurol..

[20] Munschauer F.E., Kinkel R.P. (1997). Managing side effects of interferon-β in patients with relapsing-remitting multiple sclerosis. Clin. Ther..

[21] La Mantia L., Capsoni F. (2010). Psoriasis during interferon beta treatment for multiple sclerosis. Neurol. Sci..

[22] López-Lerma I., Iranzo P., Herrero C. (2009). New-onset psoriasis in a patient treated with interferon beta-1a. Br. J. Dermatol..

[23] Ghoreschi K., Weigert C., Röcken M. (2007). Immunopathogenesis and role of T cells in psoriasis. Clin. Dermatol..

[24] Riol-Blanco L., Ordovas-Montanes J., Perro M., Naval E., Thiriot A., Alvarez D., Paust S., Wood J.N., von Andrian U.H. (2014). Nociceptive sensory neurons drive interleukin-23-mediated psoriasiform skin inflammation. Nature.

[25] Gooderham M., Posso-De Los Rios C.J., Rubio-Gomez G.A., Papp K. (2015). Interleukin-17 (IL-17) inhibitors in the treatment of plaque psoriasis: A review. Skin Ther. Lett..

[26] Axtell R.C., Raman C., Steinman L. (2011). Interferon-β exacerbates Th17-mediated inflammatory disease. Trends Immunol..

[27] Axtell R.C., Raman C. (2012). Janus-like effects of type I interferon in autoimmune diseases. Immunol. Rev..

[28] Arrue I., Saiz A., Ortiz-Romero P.L., Rodríguez-Peralto J.L. (2007). Lupus-like reaction to interferon at the injection site: report of five cases. J. Cutan. Pathol..

[29] Somani A.K., Swick A.R., Cooper K.D., McCormick T.S. (2008). Severe dermatomyositis triggered by interferon beta-1a therapy and associated with enhanced type I interferon signaling. Arch. Dermatol..

[30] Liu Z., Bethunaickan R., Huang W., Lodhi U., Solano I., Madaio M.P., Davidson A. (2011). Interferon-α accelerates murine systemic lupus erythematosus in a T cell-dependent manner. Arthritis Rheum..

[31] Hügle T., Gratzl S., Daikeler T., Frey D., Tyndall A., Walker U.A. (2009). Sclerosing skin disorders in association with multiple sclerosis. Coincidence, underlying autoimmune pathology or interferon induced?. Ann. Rheum. Dis..

[32] Kocer B., Nazliel B., Oztas M., Batur H.Z. (2009). Vitiligo and multiple sclerosis in a patient treated with interferon beta-1a: A case report. Eur. J. Neurol..

[33] Chakravarty S.D., Harris M.E., Schreiner A.M., Crow M.K. (2012). Sarcoidosis triggered by interferon-beta treatment of multiple sclerosis: A case report and focused literature review. Semin. Arthritis Rheum..

[34] Devonshire V., Arbizu T., Borre B., Lang M., Lugaresi A., Singer B., Verdun di Cantogno E., Cornelisse P. (2010). Patient-rated suitability of a novel electronic device for self-injection of subcutaneous interferon beta-1a in relapsing multiple sclerosis: An international, single-arm, multicentre, Phase IIIb study. BMC Neurol..

[35] Ghoreschi K., Brück J., Kellerer C., Deng C., Peng H., Rothfuss O., Hussain R.Z., Gocke A.R., Respa A., Glocova I. (2011). Fumarates improve psoriasis and multiple sclerosis by inducing type II dendritic cells. J. Exp. Med..

